# The Prevalence of Erosive Tooth Wear and Related Risk Factors in 6- to 12-Year-Old Students

**DOI:** 10.3290/j.ohpd.b2403635

**Published:** 2021-12-08

**Authors:** Jia-wei Liu, Xue-Ying Shi, Jia-Xin Li, Xin Li

**Affiliations:** a Master’s degree, Second Affiliated Hospital of Jinzhou Medical University, Jinzhou, Liaoning Province, China. Idea, wrote the manuscript, performed the experiments, performed statistical evaluation, experimental design.; b Master’s degree, Second Affiliated Hospital of Jinzhou Medical University, Jinzhou, Liaoning Province, China. Performed the experiments, experimental design.; c Master’s degree, Second Affiliated Hospital of Jinzhou Medical University, Jinzhou, Liaoning Province, China. Performed the experiments in partial, experimental design.; d Doctor’s degree, Professor, Second Affiliated Hospital of Jinzhou Medical University, Jinzhou, Liaoning Province, China. Proofread the manuscript, contributed substantially to discussion.

**Keywords:** erosive tooth wear, prevalence, risk factors, BEWE

## Abstract

**Purpose:**

To evaluate the epidemiological characteristics of erosive tooth wear in primary school students aged 6–12 in Jinzhou, including: prevalence, severity, extent, intraoral distribution and associated risk factors.

**Materials and Methods:**

The data collection consists of two parts: the first part is the clinical examination of the participants. All erupted teeth were clinically assessed by three calibrated examiners, and classified according to the basic erosive wear examination (BEWE); The second part is a questionnaire about demographic information, parafunctional movement and lifestyle, completed by the parents or their guardians.

**Results:**

A total of 1,469 children were included in this experiment; erosive tooth wear (ETW) prevalence (BEWE ≥ 1) was 54.9%. According to cumulative BEWE index, the proportion of different ETW severity (high, medium, low, none) was 6.8%, 16.3%, 27.0% and 49.9%, respectively. In an analysis of risk factors, family factor, age, gender, extracurricular study time, oral hygiene habit, bruxism, unilateral mastication and acid diet was associated with the prevalence of ETW.

**Conclusions:**

This study shows that ETW has a high prevalence in students aged 6–12, and more common in deciduous molars and deciduous canine. Abrasion, attrition and erosion play an important role in ETW.

The term ‘erosive tooth wear’ (ETW)^5, 16, 28^ is used to describe the loss of dental hard tissues caused by chemical-mechanical process established without bacterial involved. ETW is a multifactorial disease, in which the erosion of extrinsic or intrinsic acids and mechanical process, such as tooth wear, jointly induce this disease. The prevalence of ETW, a form of tooth wear that can be diagnosed in both primary and permanent teeth, is statistically significant. Salas et al^26^ reported that nearly one-third (30.4%) of the children have ETW in their permanent teeth, which is likely attributed to different popular nutrition elements.^27^ Generally speaking, the prevalence of ETW in primary teeth is 30–50%, and in permanent teeth is 20–45%.^29^ A survey of 11–14 school-age children in Mexico^10^ showed that the prevalence of ETW was 62.5%; a survey of 12-year-old children in Hong Kong by Zhang et al^41^ showed that the prevalence of ETW was as high as 75%; meanwhile, Mangueira et al^20^ conducted a survey in Brazil and found that the prevalence of dental erosion was 19.9%. However, the proportion of deciduous teeth was as high as 61.8%, far higher than that of permanent teeth (38.2%). Although different epidemiological surveys have different sample size, demographic information and diagnostic methods, there is no doubt that the prevalence of ETW among children and adolescents is on the rise.

First of all, erosion is the prerequisite for ETW, including intrinsic acid and extrinsic acid. Hydrochloric acid produced by gastric parietal cells is the main intrinsic factor for ETW. The presence of these intrinsic acids in the oral cavity may be due to gastroesophageal reflux disease (GERD), eating disorders, chronic vomiting or regurgitation. Gastrointestinal diseases^15, 34^ can reduce oral pH value due to frequent vomiting, regurgitation and heartburn; the extrinsic acid mainly comes from daily diet, including carbonic acid, lactic acid, acetic acid and citric acid, which are risk factors. Some disease-related acidic drugs (vitamin C, aspirin, etc) are also risk factors for ETW. Changes in dietary habits in recent years, including a higher frequency of consumption of acidic foods and drinks may have mainly contributed to that phenomenon. The acids dissolve minerals from tooth surfaces, causing a demineralised and softened surface layer which can be removed by mechanical forces easily, such as attrition and abrasion.^13, 34^ Finally, asymmetric abrasion and parafunctional habits^9^ are easy to lead to abnormal occlusion (premature contact and interference of occlusion). In mastication movement, especially lateral movement, the abfraction will be happen and cause the loss of microstructure,which is also the aetiology of ETW.

The early ETW is mainly characterised by demineralisation of enamel. When the teeth are exposed to acidic oral environment for a long time, hydrogen ion will react with calcium, phosphorus and other inorganic ions in the dental hard tissue, and the hydroxyapatite of enamel will dissolve gradually. According to a study by Eisenberg et al that compared the demineralisation of dental caries, the average depth of substance loss caused by citric acid erosion is 16 μm, and the further softening depth of enamel is 2.4 μm. Compared with the original mineral content, the content of calcium and inorganic phosphorus in demineralised enamel decreased by 38% and 36%, respectively. This zone of softened enamel has a reduced physical stability and large interprismatic porosities, which may explain why some people have less bite force, but ETW lesions progress faster.^6, 24^ The early clinical manifestations of ETW are mostly the loss of enamel texture. And sometimes, the appearance loses gloss and looks like opaque chalk. The further development of ETW will lead to the flattening of the cusp and occlusal surface, and the anatomic morphology can disappear with hollowed out surfaces. On a smooth surface, such as the palatal surface, it is usually a shallow concave defect with the width usually greater than the depth, while on the coronal side near the cementoenamel junction, it is usually a halo of enamel around the gingival margin of the crown. This may be due to the presence of dental plaque and remnants in this area can act as a barrier for acid diffusion. In addition, gingival crevicular fluid is weakly alkaline and can neutralise acid.^5^

In addition, a large number of studies have shown that there is a certain degree of correlation between ETW and demographic variables, among which family factors, gender, age and nationality are worth discussing. For example, a study from China shows that mother’s education level is an independent risk factor for ETW in children of southern China. Children whose mothers have higher levels of education show fewer lesions^37^; another survey of 5-year-old children found^34^ that high-income families and mothers of low education background were positively correlated with the severity of ETW; Luo et al’s^17^ report shows that the children whose parents have higher education background are more likely to have ETW. All in all, a better understanding of the correlation between demographic variables and the prevalence of ETW can enable us to formulate prevention and control strategies focusing more accurately on specific populations. In addition, the relationship between ETW and psychological factors and parafunctional habits will also be discussed in this article.

In China, the prevalence rate has been shown to vary from 4.5%^33^ to 27.3%.^37^ In the past decade, China’s economic development has accelerated, and Chinese lifestyle, including diet and oral hygiene habits, has significantly changed. More and more attention has been paid to dental hard tissue diseases, but the prevalence of ETW, especially in Northeast China, is still lacking.

ETW is a type of disease characterised by progressive destructiveness without obvious clinical symptoms. At the same time, the diagnosis, treatment and prevention of the disease depend on the elimination of risk factors. Therefore, the purpose of this investigation is to evaluate the prevalence, severity, extent, intraoral distribution and related risk factors of ETW in children aged 6–12 in Jinzhou, China and provide a theoretical basis for further research on dental erosion and tooth wear.

## Materials and Methods

### Study Design and Sample

According to a cross-sectional study from China, the expected prevalence rate was 60%,^16^ a precision level of 10%,a 95% confidence interval (CI), a desertion rate of 10%,^9^ thus,1,500 students would be required for the present study. Five public primary schools, including Jiqing primary school, Jiefang primary school, Shiyan primary school, Luoyang primary school and Beihu primary school, were selected by random sampling method. The epidemiological survey was approved by the ethics committee of the Second Affiliated Hospital of Jinzhou Medical University, and the parents of the children provided signed informed consent.

*Inclusion criteria*: (1) Parents and children who fully understand the purpose of the oral survey and sign the informed consent form; (2) primary school students aged 6–12 years in Jinzhou; (3) there is at least one fully erupted tooth within every sextant.

*Exclusion criteria*: (1) Those undergoing orthodontic treatment; (2) teeth that have not fully erupted; teeth with restorations that cover more than 2/3 of the tooth surface; (3) those with dental development disease such as microdontia and fluorosis.

### Clinical Exam and Questionnaire

The survey was conducted in a student classroom equipped with a portable light source, and was completed by three examiners. After cleaning the tooth surface with a cotton swab, the BEWE index^3^ was used to evaluate the ETW of each tooth. Table 1 showed the detailed criteria of BEWE index. Prevalence was defined as the percentage of individuals presenting at least one tooth with ETW (BEWE ≥ 1); all surfaces^16^ (buccal/facial, dental cervical, occlusal/incisal/cusp, and lingual/palatal) were recorded. The BEWE score of the most severely affected surface was taken as the tooth score. In addition, the most severely affected tooth in each of sextant (teeth 14–16/55–54, 13–23/53–63, 24–26/64–65, 34–36/74–75, 33–43/73–83, 44–46/84–85) were also recorded. According to Bartlett,^3^ the sum of the scores of the sextants, ranging from 0 to 18, was calculated and represented the severity of lesions (normal = 0~2, mild = 3~8, moderate = 9~13, severe = 14~18). The severity of ETW was then divided according to the sum of the highest score of each sextant.

**Table 1:** The index of BEWE

Score	
BEWE 0	No erosive tooth wear
BEWE 1	Initial loss of surface texture of dental hard tissue
BEWE 2	Surface defect of dental hard tissue, but defect <50% surface area
BEWE 3	Obvious defect, defect area >50% surface area

The parents or guardians of the participants needed to complete a questionnaire after the clinical examination. Before the final survey, we conducted a pilot study on this questionnaire. By summarising the comments of participants and experts, the questionnaire was further modified. This questionnaire was designed to investigate the related influencing factors and it included the following contents. The frequency of eating acidic foods or drinks (rarely = once to several times a month, sometimes = once a week, often = more than twice a week, usually = every day), whether to brush teeth immediately after acidic food or drink, unilateral chewing, bruxism (sleep bruxism, clenching teeth, mixed), oral hygiene habits (high, medium, low), extra study time after class (>1.5 h means yes, <1.5 h means no), demographic information: gender, nationality, age, family factors (high, medium, low) etc.

Among them, oral hygiene habits were divided into high, medium and low according to the answers of six questions, including brushing time, brushing frequency, brushing strength, bristle hardness, brushing method and whether to brush immediately after eating (Table 2). Family factors were divided into high, medium-high, medium-low and low according to the answers of the six questions about their family, including mother’s education level, residence, parents’ physical condition, the number of the elderly, the number of children and family income (Table 3).

**Table 2:** Oral hygiene habits

Oral hygiene habits questions	Score
**1. Brushing frequency**	
Never Once a day Twice a day Three times a day or more	0 2 4 6
**2. Brushing time**	
≤ 1 min 1–2 min > 2 min	2 4 6
**3. Brushing strength**	
Mild Moderate Vigorous	2 4 6
**4. Brushing method**	
Horizontal brush Vertical brush	2 4
**5. Bristle hardness**	
Soft bristles Medium Hard bristles	2 4 6
**6. Do you brush your teeth immediately after eating**	
Yes No	2 4

Low: 12–18, Medium: 19–25, High: 26–32

**Table 3:** Family factors

Family factors questions	Score
**1. Parents’ education level**	
Junior college Under graduate Postgraduate	2 4 6
**2. Residence**	
Rural City	2 4
**3. Disposable income**	
All < ¥ 30733 (per capita disposable income in China) All ≥ ¥ 30733 One > ¥ 30733, the other < ¥ 30733	2 6 4
**4. Physical condition of parents**	
All healthy Both parents are ill and in poor health One is healthy, the other is in poor health due to illness	6 2 4
**5. Supporting the elderly**	
One Two More than two	6 4 2
**6. Number of children**	
One Two More than two	6 4 2

Low: 12–17, Medium-low: 18–23, Medium-high: 24–29, High: 30–34

### Statistical Analysis

Statistical analysis of the collected data was performed using SPSS22.0 for descriptive statistical analysis, using Pearson’s chi-square test for bivariate analysis, and variables with statistical significance were further included in the binary logistic regression analysis and calculate the odds ratio (OR) and 95% CI, so as to distinguish risk factors. The potential confounders are adjusted according to sociodemographic information (gender, age, nationality, family-social factors).^31^ In this part, we will carry out two groups of statistical analysis, and the discussion in this paper was based on statistical analysis results of the first group.

*First group*: The prevalence of ETW (BEWE>1) was set as the dependent variable. Chi-square test and binary logistic regression analysis were performed to determine the influencing factors related to the prevalence of ETW.

*Second group*: The severity of ETW (the cumulative BEWE was 9–18) was set as the dependent variable. Chi-square test and binary logistic regression analysis were also used to determine the influencing factors related to medium–high risk ETW. The statistically significant level was set to *P* <0.05.

### Reliability Test

In the early stage of the epidemiological investigation, all three examiners received clinical training about ETW. The reliability of interexaminer and intraexaminer was evaluated before the final investigation by means of reliability test in 20 selected participants (including a reference examiner and three examiners). The values of the intraexaminer kappa coefficient were 0.90, 0.92 and 0.87, and the interexaminer kappa value was 0.80. In the middle of the investigation, 5% of the subjects were selected for the second test of reliability between the examiners, and the kappa coefficient values were all greater than 0.85.

## Results

### Participants’ Information

Out of a total 1,500 participants, 11 students did not complete the questionnaire survey, 4 students’ parents did not sign informed consent, and 16 students were excluded because they did not meet the inclusion criteria. So 1,469 participants underwent a complete clinical examination and questionnaire survey, while the participation rate was 97.9%. This survey found that 806 students had ETW (at least one tooth BEWE ≥ 1), and out of 475 boys and 331 girls, the overall prevalence of ETW was 54.9% (95%CI: 52.3–57.4).

### Severity and Distribution

**Fig 1:**
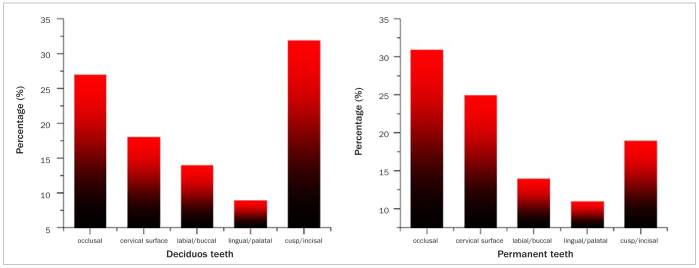
Distribution of individual teeth by highest basic erosive wear examination score in different tooth surfaces.

**Fig 2:**
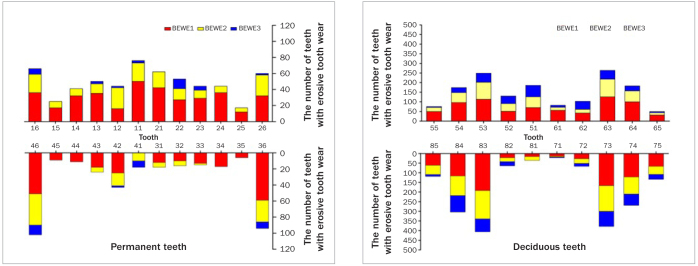
Distribution of individual teeth by basic erosive wear examination score in different tooth.

Regarding the severity of ETW lesions, 100 individuals had severe ETW (14–18), 240 individuals had moderate ETW (9–13), 397 individuals had mild ETW (3–8), and 69 individuals had normal condition (0–2). The distribution of tooth surface with BEWE3 represented ETW extent. In deciduous teeth, BEWE3 mostly occurred on the occlusal surface and cusp. In permanent teeth, BEWE3 mostly occurred on the occlusal surface and cervical surface. The individual distribution of the highest BEWE scores on different tooth surfaces was shown in Figure 1. Figure 2 showed the intraoral distribution characteristics of ETW, in which primary canines were the most commonly affected teeth, followed by primary molars. In permanent teeth, ETW was more common in the first molar and maxillary anterior teeth.

### Risk Factors of ETW Prevalence

Table 4 showed the frequency distribution of demographic variables. We found a statistically significant correlation between demographic variables and the ETW prevalence by the chi-square test. Those who were male, had higher family factors, belonged to minorities, and were 6–9 years old had higher prevalence of ETW. Table 5 showed the frequency distribution of different influencing factors. Those who ate acidic food usually, had higher oral hygiene habit, had bruxism, had unilateral mastication habit and had longer extra study time had a higher prevalence of ETW. Table 6 showed the results of binary logistic regression analysis. We used OR = 1 as standard to distinguish risk factors. In addition to unilateral mastication (Adj OR: 1.082; *P* >0.05) and minority (Adj OR: 0.465; *P* <0.05), other variables can be regarded as independent risk factors for ETW prevalence (Adj OR >1; *P* <0.05).

**Table 4:** Frequency distribution between demographic factors and the prevalence of ETW (chi-square test)

Variables	N N = 1469	Erosive tooth wear	X^2^	*P*
		BEWE ≥ 1 n%		
**Age**				
6–9 years 10–12 years	739 730	525 (71.0%) 281 (38.5%)	157.122	*P < 0.05*
**Gender**				
Male Female	722 747	475 (65.8%) 331 (44.3%)	68.400	*P < 0.05*
**Family-social factors**				
Low Medium–low Medium–high High	322 340 427 380	101 (31.4%) 161 (47.4%) 309 (72.4%) 235 (61.8%)	139.830	*P < 0.05*
**Nationality**				
Han Minority	1051 418	547 (52.2%) 259 (62.0%)	11.875	*P < 0.05*

**Table 5:** The percentage of participants with erosive tooth wear according to different influencing factors (chi-square test)

Variables	N N = 1469	Erosive tooth wear	X^2^	*P*
		BEWE ≥ 1 n%		
**Oral hygiene habits**				
High Medium Low	602 529 338	379 (63.0%) 302 (57.1%) 125 (37.0%)	60.624	*P < 0.05*
**Frequency of eating acid flavouring**				
Rarely Sometimes Often Usually	326 368 395 380	114 (35.0%) 177 (48.9%) 239 (60.5%) 276 (72.6%)	112.431	*P < 0.05*
**Frequency of eating sauerkraut**				
Rarely Sometimes Often Usually	298 316 411 444	101 (33.9%) 133 (42.0%) 255 (62.0%) 317 (71.4%)	131.315	*P < 0.05*
**Frequency of drinking soft drinks**				
Rarely Sometimes Often Usually	328 441 476 224	130 (39.6%) 240 (54.4%) 298 (62.6%) 1387 (61.6%)	11.875	*P < 0.05*
**Unilateral mastication**				
Yes No	486 983	296 (60.9%) 510 (51.9%)	10.693	*P < 0.05*
**Extra study time**				
< 1.5 h ≥ 1.5 h	897 572	418 (46.6%) 388 (67.8%)	63.586	*P < 0.05*
**Bruxism**				
Sleep bruxism Clench Mixed Normal	143 221 198 907	99 (69.2%) 136 (61.5%) 157 (79.3%) 414 (45.6%)	94.741	*P < 0.05*
**Brush teeth immediately after an acidic diet**				
Yes No	245 1,224	135 (55.1%) 671 (54.8%)	0.007	*P < 0.05*

**Table 6:** The relation between risk factors and the prevalence of ETW according to logistic regression model (crude: OR and adjust OR)

Variables	*P:*	Crude OR:	Adjust OR:	95%CI:
**Age**				
10–12 6–9	*P < 0.05*		1 4.987	3.736–6.658
**Gender**				
Girl Boy	*P < 0.05*		1 1.886	1.425–2.497
**Nationality**				
Han Minority	*P < 0.05*		1 0.465	0.338–0.640
**Family-social factors**				
Low Medium-low Medium-high High	*P < 0.05* *P < 0.05* *P < 0.05* *P < 0.05*		1 2.852 7.829 4.346	1 1.875–4.338 5.145–11.914 2.873–6.574
**Oral hygiene habits**				
Low Medium High	*P < 0.05* *P < 0.05* *P < 0.05*	1 2.855 3.308	1 3.070 4.099	1 2.096–4.495 2.792–6.019
**Frequency of eating acid flavouring**				
Rarely Sometimes Often Usually	*P < 0.05* *P < 0.05* *P < 0.05* *P < 0.05*	1 1.816 2.714 5.100	1 1.734 2.926 5.468	1 1.152–2.612 1.967–4.353 3.597–8.311
**Frequency of eating sauerkraut**				
Rarely Sometimes Often Usually	*P < 0.05* *P < 0.05* *P < 0.05* *P < 0.05*	1 1.456 2.646 4.959	1 1.642 3.395 6.309	1 1.079–2.500 2.256–5.109 4.133–9.630
**Frequency of drinking soft drinks**				
Rarely Sometimes Often Usually	*P < 0.05* *P < 0.05* *P < 0.05* *P < 0.05*	1 1.833 2.192 2.196	1 2.015 2.394 2.426	1 1.374–2.956 1.618–3.544 1.523–3.864
**Unilateral mastication**				
No Yes	*P > 0.05*	1 1.098	1 1.082	1 0.804–1.455
**Extra study time**				
No Yes	*P < 0.05*	1 2.621	1 2.746	1 2.052–3.675
**Bruxism**				
Normal Sleep bruxism Clenching Mixed	*P < 0.05* *P < 0.05* *P < 0.05* *P < 0.05*	1 1.766 2.089 4.456	1 1.775 1.802 4.532	1 1.095–2.876 1.205–2.696 2.866–7.167

### Risk Factors of ETW Severity

According to Table 7, ETW severity was associated with the frequency of eating acid foods, bruxism, age, gender, family factors, oral hygiene habits and extra study time. According to Table 8, age, gender, family factors, oral hygiene habits (high), eating acid flavouring (usually), eating sauerkraut (often, usually), drinking soft drinks (usually), and extra study time (yes) can be considered as risk factors of ETW severity (Adj OR>1; *P* <0.05).

**Table 7:** Chi-square test with ETW severity as dependent variable

Variables	X^2^	*P* value
**Age**	15.635	*P < 0.05*
**Gender**	26.873	*P < 0.05*
**Family factors**	50.766	*P < 0.05*
**Nationality**	3.302	*P > 0.05*
**Oral hygiene habits**	14.543	*P < 0.05*
**Frequency of eating acid flavouring**	26.364	*P < 0.05*
**Frequency of eating sauerkraut**	23.616	*P < 0.05*
**Frequency of drinking soft drinks**	17.000	*P < 0.05*
**Unilateral mastication**	0.005	*P > 0.05*
**Extra study time**	18.183	*P < 0.05*
**Bruxism**	12.341	*P < 0.05*
**Brush teeth immediately after an acidic diet**	0.378	*P > 0.05*

**Table 8:** The results of the binary logistic regression analysis with ETW severity as the dependent variable

Variables	*P* value	OR:	95%CI:
**Age**			
10–12 6–9	*P < 0.05*	1.451 1	1.112–1.893
**Gender**			
Girl Boy	*P < 0.05*	1.538 1	1.181–2.002
**Family factors**			
Low Medium-low Medium-high High	*P < 0.05* *P < 0.05* *P < 0.05*	1 1.834 3.433 2.370	1.167–2.881 2.267–5.198 1.543–3.641
**Oral hygiene habits**			
Low Medium High	*P > 0.05* *P < 0.05*	1 1.282 1.773	0.884–1.858 1.232–2.538
**Frequency of eating acid flavouring**			
Rarely Sometimes Often Usually	*P > 0.05* *P > 0.05* *P < 0.05*	1 1.065 1.295 1.789	0.704–1.611 0.872–1.924 1.216–2.633
**Frequency of eating sauerkraut**			
Rarely Sometimes Often Usually	*P > 0.05* *P < 0.05* *P < 0.05*	1 1.277 1.741 1.796	0.820–1.989 1.159–2.651 1.205–2.677
**Frequency of drinking soft drinks**			
Rarely Sometimes Often Usually	*P > 0.05* *P > 0.05* *P < 0.05*	1 1.637 1.573 1.955	1.105–2.424 1.073–2.306 1.259–3.037
**Extra study time**			
Yes No	*P < 0.05*	1.553 1	1.197–2.016
**Bruxism**			
Sleep bruxism Clenching teeth Mixed Normal	*P > 0.05* *P > 0.05* *P > 0.05*	1.085 1.156 1.388 1	0.708–1.665 0.800–1.670 0.964–1.998

## Discussion

This population-based cross-sectional study found that the prevalence of ETW in children aged 6–12 years in Jinzhou, China, was relatively high. There were 1,469 students, of which 806 students were found to have ETW in the oral cavity (BEWE≥ 1) However, as far as the severity of the lesion was concerned, only 6.8% individuals had severe ETW, and the most of individuals had mild ETW. That is to say, ETW is more common in some susceptible teeth but not all teeth, such as primary molars, first molars and canines. This is because the occlusal surface of the molar is the main functional surface of masticatory movement. At the same time, the primary canines are in the turning point of dental arch, which are easy to form stress concentration and be eroded by exogenous acid. However, the prevalence of ETW in the remaining teeth of the oral cavity is at a low level because of special anatomic and functional factors. For example, the maxillary posterior teeth are located at the opening of salivary duct, such as parotid gland, which leads to lower prevalence of these teeth. Even if some susceptible teeth have more serious ETW, the severity of ETW, according to cumulative BEWE index, is still low. On the other hand, the severity of ETW in 69 participants was classified as normal condition, because these patients with cumulative BEWE index in the range of 1–2 were not considered to have ETW risk.

Based on the analysis of the survey data, we found that the prevalence of ETW among children aged 6–12 years in Jinzhou was 54.9%, which was similar to the ETW survey in Brazilian teenagers (57%),^23^ ETW survey in Guangzhou (56.1%),^16^ and the ETW survey of children aged 6–12 in Mexico (62%).^9^ However, a study on 12-year-old teenagers in the central city of Wuhan^40^ showed that the prevalence of tooth wear was 18.6%. The difference in results may be attributed to different diagnostic criteria. They diagnosed teeth with BEWE≥2 as ETW. Although different epidemiological investigations use different clinical examination methods, diagnostic standards, and sample sizes, it is undeniable that the prevalence of ETW is gradually increasing worldwide, but people’s awareness of such diseases needs to be improved. Hu et al^39^ conducted a survey on the degree of understanding of dental erosion among dental clinic patients and found that 76% of the population had never heard of dental erosion, and only 45% believed that acidic beverages would cause chronic damage to teeth. A survey in Norway^34^ also demonstrated that a high proportion of students lacked basic knowledge of what ETW was. This is similar to the results of our questionnaire survey – 69% of people are unaware of chronic tooth damage, and only 21% think that acid will cause chronic damage to teeth; this indicates that our knowledge about dental hard tissue lesions is obviously insufficient, the prevention and healthcare of related oral diseases still needs further development.

Whether there is a correlation between family factors and the prevalence of ETW is still controversial. In this survey, we divided family factors into high, medium–high, medium–low and low based on different family backgrounds. The results show that the difference between family factor and the prevalence of ETW is statistically significant (P <0.05), which is similar to the result of Débora Nunes de Oliveira et al.^23^ They suggested that there is a statistically significant correlation between socioeconomic status and the prevalence of ETW, and this correlation is caused by a combination of multiple factors, not only economic factors. In the binary logistic regression model, we found that there was a clear correlation between the family factor of medium–high and the prevalence of ETW (Adj OR: 7.829; 95%CI: 5.145–11.914). These families,^23^ to a certain extent, not only have a level of economic strength and cognitive level, but also have a limited amount of time to pay attention to their children’s oral health, so that the children of these families have lower sugar consumption, better oral hygiene habits, and low dental caries prevalence.^7^ And those children with lower levels of family factors have a lower prevalence of ETW. This may be related to the prevalence of dental caries and family economic situation. On the one hand, children with lower family factors generally have higher caries prevalence, which affects the diagnosis of ETW. In addition, the diet of these children is relatively simple. Another survey from Brazil^1^ also found that there was a correlation between the prevalence of ETW and middle-income families.

Regarding demographic variables, our study found a correlation between gender and the prevalence of ETW (Adj OR: 1.886; 95% CI: 1.425–2.497). The prevalence of ETW in boys and girls was 65.8% and 44.3%, respectively, and the difference was statistically significant. This is similar to previous studies,^10, 23, 34, 41^ which can be attributed to differences in living habits, eating habits and physiological factors. For example, boys exercise a high frequency. According to the questionnaire, boys spend three times more outdoor exercise per week than girls. After exercise, the amount of saliva secretion decreases due to the loss of body fluid. The decrease of velocity of flow and flow rate can cause the decrease of buffering capacity and remineralisation ability, which increases the risk of ETW.^22^ At the same time, the phenomenon of drinking beverages immediately after exercise is also more common in boys, which leads to gender differences. In addition, boys have higher bite force than girls, which accelerates the development of ETW to a certain extent. Finally, boys prefer acidic beverages to girls.^34^ This survey also found that among the people who drink acidic beverages frequently, the sex ratio of men to women is 3:1.

As for nationality factors, de Oliveira et al^23^ investigated the differences in the prevalence of ETW among people of different skin colours in Brazil, and found that there are differences between race and ETW. The report released in 2011^1^ also found the same conclusion. In China, the Han population accounts for 91.11% of the total population, and the minorities account for 8.89%. All nationalities except Han are defined as minorities, so our experiment divides the nationality factors into Han Chinese and minority Chinese, that is, non-Han Chinese. Our survey included 1,050 Han students and 419 minority students. Although there was statistical significance between nationality and ETW prevalence (P <0.05), binary regression analysis showed that compared with the Han nationality, the minority factor was a protective factor (Adj OR: 0.465; 95%CI: 0.338–0.640). Nationality is considered as a demographic variable in epidemiological survey because there are differences in family socioeconomic conditions among different nationalities. However, with the development of society, the imbalance of economic development gradually disappears. And it is also believed that the differences in dietary structure between nationalities are the reasons for the different prevalence of ETW. The Han nationality’s diet is soft, while the minority people such as Manchu and Mongolian have a harder diet, which often requires greater chewing power, and can easily lead to unilateral mastication. However, this survey found that there is no statistically significant difference in eating habits between nationalities.

**Fig 3:**
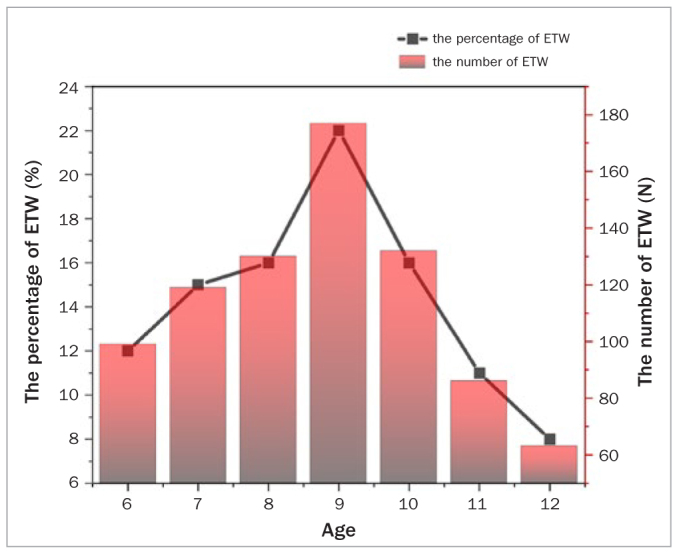
The percentage and number of ETW in different ages.

The increase of age means the increase of the prevalence rate, which is the common feature of non-carious lesions of dental hard tissue. The epidemiological surveys of ETW in Chile^21^ and Wuhan^40^ have reached consistent conclusions. However, in the mixed dentition period, due to the replacement of deciduous and permanent teeth, the prevalence of ETW with age presents a special trend. An ETW survey of children aged 6–12 in Mexico^9^ showed that dental erosion was mostly concentrated in the 6–10 age group. Our survey found that the prevalence of ETW is as high as 71% in the 6–9 age group, and only 38.5% in the 10–12 age group. Deciduous teeth, compared with their permanent counterparts, are generally smaller and have a thinner enamel layer. Besides the anatomical factors, there are additional histological differences that may influence ETW prevalence. Firstly, in relation to the enamel crystals, the primary and permanent teeth are similar, but the arrangement of the enamel prims of the primary teeth is more curved, smaller and more widely distributed. This indicates that the enamel of the primary teeth is more porous than that of the permanent teeth. Secondly, the organic content of enamel in deciduous teeth is 0.7–12%, but the organic content of enamel in permanent teeth is only 0.4–0.8%.^32^ All these above-mentioned differences between deciduous and permanent enamel may be related to the fact that the deciduous teeth are more susceptible to dissolution than permanent teeth.^4^ After the age of 10, deciduous molars and deciduous canines, which are prone to ETW, were replaced by permanent teeth, resulting in a decrease in the prevalence.^8^ The risk of ETW was statistically significantly increased in 6–9-year-old students compared with 10–12-year-old students (Adj OR: 4.987; 95%CI: 3.736–6.658). In the 6–9-year-old age group, the prevalence of ETW gradually increased with age, while the 10–12-year-old age group showed a downward trend (Fig 3). However, with the prolonged exposure time of young permanent teeth to oral environment, the prevalence of ETW in corresponding teeth will increase. According to an epidemiological survey in China,^40^ the prevalence of tooth wear among 12-year-old children was 18.9%, while that of 15-year-old children rose to 89.4%, suggesting that ETW is a time-dependent disease.^34^

The correlation between oral hygiene habits and the prevalence of ETW is controversial.^34,40^ Our investigation found that the difference between different oral hygiene habits and the prevalence of ETW is statistically significant (P < 0.05). The regression analysis shows that the risk of ETW in children with good oral hygiene habits was 4.099 times higher than that in children with poor oral hygiene habits (Adj OR: 4.099; 95% CI: 2.792–6.019). The reasons may be: firstly, the longer the brushing time, the higher the brushing frequency, and the harder the brushing, the better the condition of oral hygiene, however, these kind of oral hygiene habits also accelerate the loss of dental hard tissue to a certain extent, which are called attrition. Secondly, there exist acquired enamel pellicle^25^ formed by proteins, lipids and carbohydrates on the surface of teeth, which is an acellular biofilm. When acidic substances contact with dental enamel, they are first blocked by the acquired enamel pellicle. However, brushing teeth will remove these protective layers mechanically. Finally, people with poor oral hygiene habits have more dental caries,^41^ and the prevalence of caries is much higher than ETW. Alvarez-Arenal et al^2^ also found the correlation between tooth brushing strength and ETW. However, Zhang et al^41^ concluded to the contrary, the higher the prevalence of ETW in patients with short brushing time, because children or adolescents like sweet and sour food. When brushing for a short time, the food residue on the tooth surface, together with bacteria, leukocytes and some exfoliated epithelial cells, forms dental plaque, in which bacteria decompose organic substances to produce acid and further erodes teeth and finally leads to ETW.

This study found that there is a correlation between the frequency of acidic food intake and the prevalence of ETW. Due to changes in eating habits, the frequency of acidic food intake has increased, and the PH value of the oral microenvironment has been continuously reduced. People in Northeast China like to eat sauerkraut, a kind of Chinese cabbage pickled with salt. The survey found that people who consume sauerkraut accounted for 87% of the total number of people surveyed. Vegetables are generally fermented by salt and vinegar with lactic acid bacteria with a pH of 3.2–3.6, which has a statistically significant erosive effect on tooth. Compared with those who rarely eat such foods, the risk of ETW is 6.309 times higher in those who usually eat sauerkraut (Adj OR: 6.309; 95% CI: 4.133–9.630). In addition, the high frequency of intake of vinegar, chili sauce, salad dressing, ketchup and other flavouring^2,10^ is also strongly correlated with the prevalence of ETW (Adj OR: 5.468; 95%CI: 3.597–8.311). For example, a Swiss study investigates the erosive potential of bottled salad dressings. And the results show that some bottled dressings have erosive potential even higher than orange juice, especially those with low calcium content.^11^

The drinking frequency of acidic beverage was also closely related to ETW. The risk of ETW was 2.426 times higher in those who usually drank acid drinks than those who drink acid drinks rarely (Adj OR:2.426; 95%CI:1.523–3.864). Due to the existence of various acidic substances in acidic drinks, such as carbonic acid, citric acid, tartaric acid, etc, the pH value is as low as 2.5. So, the erosive effect of carbonated beverage on the enamel surface is evident. When the experimental enamel blocks was soaked in acid drinks, the surface microhardness reduced markedly and found highly etched appearance.^19^ However, the potential harm of different acid beverages to teeth is also different. For example, yogurt and probiotic beverages are not easy to cause erosion. This may be related to the content of calcium and phosphate ions in the beverage. Research by Thiago Saad Carvalho et al^25^ showed that the de- and remineralisation of dental hard tissue is mediated by continuous ion exchange between enamel surface and oral environment. Under normal circumstances, enamel and surrounding saliva are rich in (Ca^2+^, PO4^3–^, OH^–^, F^–^, CO3^2–^, Na^+^), so they are in constant balance. However, when the surrounding liquid lacks Ca^2+^ and PO4^3–^ and rich in H^+^ (erosive process), the H^+^ in the oral environment will react with the enamel surface, and the equilibrium state will be destroyed. With the dissolution of Ca^2+^, PO4^3–^ and HO^–^ from the enamel into the surrounding liquid, the ion balance state will gradually recover, in other words, this is demineralisation. Therefore, we speculate that acidic drinks rich in calcium and phosphate ions are beneficial to the ion balance of oral environment and can effectively inhibit demineralisation. As one study shows that if calcium and phosphate are added to orange juice, when the pH value is 4, the enamel is still not eroded.^14^ Our experimental survey found that 15.2% of people drink soft drinks usually, but there are still some people who do not suffer from ETW. Although it may seem evident that individuals who frequently expose their teeth to acids are at high risk of having ETW, some studies have shown that, despite the risk, not all patients display erosive lesions.^35^ Therefore, further investigation is needed to explore the impact of different brands, different composition of acidic beverages and different ways of drinking on the prevalence of ETW.

Bruxism can be divided into sleep bruxism, clenching and mixed type. It is manifested as unconsciously clenching teeth during the day or grinding teeth after falling asleep at night. In children, the clenching type also manifests as unconsciously biting pencils and nails. These parafunctional habits will lead to masticatory muscles continuing to contract and produce a greater bite force, which is not conducive to the health of dental hard tissue. The diagnosis of bruxism^38^ is clinically divided into two methods: one is accurate diagnosis, which is confirmed by electromyography; and the other is possibility diagnosis, which is through self-report. However, for the diagnosis of bruxism, the second method is often adopted. In view of the limitations of self-reporting, we collect information on bruxism in two ways. First of all, in the clinical examination stage, we ask the participants three questions,^36^ including: When you are awake, will you grind your teeth or clench your jaws? Did someone mention or are you aware yourself that you grind your teeth or clench your jaws during sleep? During the day, do you bite hard objects such as pencils unconsciously? In addition, we used questionnaires to ask the parents of the subjects about their children’s bruxism. Both parties answered ‘yes’, and were diagnosed with bruxism. The prevalence was 38.2%.

Bruxism has a clear correlation with mental and psychological abnormalities. Negative emotions, tension, fatigue, etc. will cause part of the cerebral cortex to be in a state of continuous excitement and trigger parafunctional movement. Our survey found that more than half of the students who have spent a long time in extracurricular study also have bruxism, which shows that academic burden is still the main source of pressure on contemporary students, meanwhile, mental and psychological pressure is the main precipitating factor of bruxism. There is a correlation between different types of bruxism and ETW, among which mixed bruxism is the highest (Adjqq’118ing, OR: 4.532; 95% CI: 2.866–7.167). In addition, more than 80% of the subjects with bruxism also have acid dietary habits. On the one hand, the dental hard tissue demineralises and softens under the effect of exogenous acid; on the other hand, physical effects such as bruxism and clenching aggravate the damage process, which is more common in the incisors of anterior teeth and occlusal surfaces of posterior teeth.

Unilateral chewing is also common in the population.^40^ Among participants with unilateral chewing, 60.9% also have ETW. Compared with normal chewing, unilateral chewing can be considered as a risk factor for ETW, but it is not statistically significant (*P* >0.05). First of all, the cause of unilateral chewing may be caries or missing teeth on one side, which reduces the detection rate of ETW. The survey also found that more than half of the patients with unilateral chewing had caries or missing teeth. Secondly, preference for eating harder foods, unconscious behaviours, etc. may lead to unilateral chewing and lead to excessive tooth wear on the chewing side. However, due to the fact that the information mainly depends on the questionnaire survey, it is subjective and prone to recall bias.

It is worth discussing whether brushing teeth immediately or delaying tooth brushing after eating acidic foods. Chinese study shows that^39^ after immersing in saliva for 10 min, the isolated teeth will still leave slight scratches on the surface (simulated brushing), but after 30 min of immersion, the scratches almost disappeared. Shahbaz et al^30^ also showed that brushing teeth immediately after drinking carbonated beverages or juices showed a high risk of ETW. However, our survey found that the difference between the habit of brushing immediately and the prevalence of ETW was not statistically significant (*P* >0.05). A meta-analysis^12^ also showed that the theory of delayed brushing after an acidic diet is based on the potential remineralisation of saliva, but the remineralisation effect of saliva is not only small, but also a slow process, and it only occurs on the surface of the lesion. Even if exposed to saliva for more than 240 min, the softened enamel surface is still weak in mechanical properties, and it is difficult to resist mechanical stimuli such as abrasion. In addition, the amorphous mineral deposition on the surface of teeth may not be the most ideal remineralisation form when enamel is exposed to saliva after contact with acidic substances. Lussi et al^18^ investigated the effect of immediate or delayed brushing (30 min, 120 min and 240 min) on ETW after tooth etching, and reached the same conclusion as our experiment.

Finally, we found through a questionnaire that 65% of children spend more than 3 h of extra study time per day, and 59% of children spend more than 6 h studying on weekends. Longer extracurricular study time can be regarded as a risk factor of ETW (Adj OR: 2.746; 95%CI: 2.052–3.675). Long extracurricular learning is a kind of spiritual burden for children. The questionnaire survey found that most students think that extracurricular learning content is more difficult, especially boys. And the difference between the learning time and whether existing parafunctional movement is statistically significant and positively correlated (Chi-squared test *P* = 0.698). Therefore, psychological issues are still a risk factor that cannot be ignored in non-carious dental lesions.

### Limitation

This study is a cross-sectional study, people’s occlusal habits or eating habits are not fixed, but develop longitudinally. Therefore, in order to obtain more accurate data, further longitudinal research methods should be adopted. Secondly, this experiment used questionnaires to obtain information on bruxism, unilateral chewing, etc, with a certain degree of subjectivity. In addition, this experiment uses the BEWE index to assess the prevalence of ETW, but lacks standardization to determine the depth of the lesion (whether it involves dentin or only confined to enamel), which is a key information for the clinical treatment. Finally, it is demonstrated that genetic predisposition, as well as biological factors of the host, that is, salivary flow and composition, and dental pellicle, also have an important role in the multifactorial aetiology of ETW. Thus, the relationship between genetic susceptibility and environmental factors needs further research.

## Conclusion

This population-based cross-sectional survey shows that the prevalence of ETW (BEWE≥1) in children aged 6–12 years in Jinzhou, China is high (54.9%). The severity of lesions (high, medium, low, none) was 6.8%, 16.3%, 27.0% and 49.9%, respectively. In terms of intraoral distribution, ETW are more common in deciduous teeth than young permanent teeth, mostly in occlusal surface, cusp and dental cervix. Age (6–9 years), gender (boy), family factors (medium–high, high), oral hygiene habits (medium, high), frequency of eating acidic flavouring (sometimes, often, usually), sauerkraut (sometimes, often, usually), acid beverage (often), extracurricular study time (>1.5 h), bruxism (mixed type, clenching, sleep bruxism) can be regarded as risk factors.
